# Overlap Syndrome Involving Systemic Lupus Erythematosus and Autoimmune Hepatitis in Children: A Case Report and Literature Review

**DOI:** 10.3389/fped.2019.00310

**Published:** 2019-07-31

**Authors:** Wan-Tz Lai, Wan-Hua Cho, Hock-Liew Eng, Ming-Hui Kuo, Fu-Chen Huang

**Affiliations:** ^1^Department of Pediatrics, Kaohsiung Chang Gung Memorial Hospital, Chang Gung University College of Medicine, Kaohsiung, Taiwan; ^2^Department of Ophthalmology, Kaohsiung Chang Gung Memorial Hospital, Chang Gung University College of Medicine, Kaohsiung, Taiwan; ^3^Department of Anatomic Pathology, Kaohsiung Chang Gung Memorial Hospital, Chang Gung University College of Medicine, Kaohsiung, Taiwan

**Keywords:** overlap syndrome, childhood, systemic lupus erythematosus, autoimmune hepatitis, jaundice

## Abstract

**Background:** The diagnosis of overlap syndrome involving systemic lupus erythematosus (SLE) and autoimmune hepatitis (AIH) is not easily established because of its similar clinical presentations and biochemical features to those of lupus hepatitis. The term overlap syndrome is usually used in the context of overlap of autoimmune hepatitis with PSC (primary sclerosing cholangitis) or PBC (primary biliary cholangitis). Few cases of AIH complicated by SLE have been reported in the literature, and the condition is even rarer in childhood.

**Case presentation:** Here we report the case of a 16-year-old girl with SLE who initially presented with autoimmune (cholestatic) hepatitis. According to American Association for the Study of Liver Diseases practice guidelines, the diagnosis was made based on aggregated scores including female (+2); ALP:AST (or ALT) ratio <1.5(+2); elevated serum IgG level(+3); ANA > 1:80 (+3); negative hepatitis viral markers and drug history (+3, +1); average alcohol intake <25 g/day (+2); and histological interface hepatitis features (+3). She then developed a malar rash, ANA positivity, anti-double-stranded DNA (anti-dsDNA) antibodies, and a low complement level. She met 4 of 17 Systemic Lupus International Collaborating Clinics classification criteria (1) for SLE. Our patient responded very well to corticosteroid at an initial dose of methylprednisolone 40 mg Q12H for 4 days tapering to 1 mg/kg/day according to liver function test results and bilirubin level. No relapse occurred during the 3-year follow-up course.

**Conclusions:** Overlapping of SLE and AIH should be suspected when children with SLE have impaired liver function or AIH patients present with a malar or other skin rash. Liver biopsy plays an important role in establishing the differential diagnosis of SLE with liver impairment or overlap with AIH. The prompt diagnosis and adequate further treatment plans can improve disease outcomes.

## Background

Systemic lupus erythematosus (SLE) is a systemic autoimmune disease characterized by multiple clinical features and autoantibody series; its diagnostic criteria have been modified over the past 40 years with discoveries regarding its pathophysiology and clinical nature (2) SLE affects the central nervous system, cardiovascular system, lungs, liver, kidneys, joints, and skin (3).

Autoimmune hepatitis (AIH) presents as generally unresolved inflammation of the liver of unknown cause and it is characterized by autoantibodies, hypergammaglobulinemia, and interface hepatitis on histological examination. The diagnosis of AIH is based on characteristic clinical and laboratory findings, abnormal serum globulin levels, the presence of one or more characteristic autoantibodies, and histologic abnormalities (4, 5).

AIH–SLE overlap syndrome has not been clearly distinguished from lupus hepatitis due to similarities in their clinical and biochemical features, and few cases of AIH complicated with SLE have been reported. Due to the rarity of the condition, its exact frequency is unclear (6). Here we report a case of a patient who presented to our hospital with elevated liver enzymes followed by jaundice 1 month later and was later found to have AIH and underlying SLE with no viral or drug-related etiology.

## Case Presentation

A 16-year-old girl was admitted to our ward in October 2015 complaining of an icteric scleral appearance for 1 week. Accompanying symptoms included intermittent dull abdominal pain over the right upper abdomen and tea-colored urine. Tracing back her history, elevated liver enzymes (aspartate aminotransferase [AST], 397 U/L and alanine aminotransferase [ALT], 437 U/L) were noticed on the routine health examination for junior high school students 1 month before presenting with jaundice. She had no history of alcohol abuse, viral infections, or exposure to blood products. No fever, nausea or vomiting, or clay-colored stool was observed during this period, and no specific medication was taken. She had no new dietary habits, recent travel history, or contact with sick individuals. There was no known systemic or hereditary disease. A history of rash after taking an unspecified painkiller was mentioned.

Upon arriving at our emergency department, her vital signs were as follows: body temperature, 36.5°C; pulse, 90/min; respiratory rate, 20/min; blood pressure, 115/68 mmHg; and saturation, 100% on room air. Physically, she looked normal at the time when the icteric sclera were observed. No lymphadenopathy or neck mass was palpated. The chest expanded symmetrically and the breathing sounds were clear; her heart sounds were regular without an audible murmur; the abdomen was soft and the bowel sounds were normal while mild dull pain was noted over the right upper quadrant. No cyanosis or pitting edema over the extremities was noted. Laboratory tests revealed no leukocytosis (white blood cell count, 5,400/μL) but anemia (hemoglobin, 10.5 g/dL). Elevated direct bilirubin (8.69 mg/dL), total bilirubin (11.6 mg/dL), AST (931 U/L), ALT (507 U/L), hypoalbuminemia (2.8 g/dL), gamma-glutamyl transpeptidase (GGT) (23 U/L), alkaline phosphatase (136 U/L), prolonged prothrombin time (PT; 14.8 s), and activate partial thromboplastin time (aPTT; 36 s) were also detected. Under the impression of cholestatic hepatitis, she was admitted for further observation and treatment.

After admission, ursodeoxycholic acid (100 mg 1pc QID) was administered to treat the cholestatic hepatitis. The cause of the cholestatic hepatitis was considered: anti-hepatitis A virus immunoglobulin M (IgM), anti-hepatitis B core antigen IgM, hepatitis B surface antigen (enzyme-linked immunosorbent assay), anti-hepatitis C virus antibody (Ab), Epstein-Barr virus/cytomegalovirus immunoglobulin G (IgG)/IgM, herpes simplex virus for viral hepatitis were negative. Survey for Wilson's disease included serum ceruloplasmin (21.40 mg/dL) and copper (101.9 μg) were within normal value. Abdominal ultrasonography revealed a thick gallbladder wall (0.5 cm) with pericholecystic fluid accumulation, which may be related to hepatitis. The bilirubin level elevated despite of ursodeoxycholic acid usage. A follow-up liver function test 3 days later revealed total bilirubin, 15 mg/dL (direct, 10.96 mg/dL); AST/ALT, 1123/553 U/L; GGT, 23 U/L; and albumin, 3.0 g/dL. We also checked her serum iron level to rule out hemochromatosis; an elevated iron level (212 μg/dL) was found, while total iron binding capacity (TIBC) and the serum lead concentration were in the normal ranges. Tests for hemolysis, including direct and indirect Coombs' test, peripheral blood (PB) smear, red blood cell (RBC) fragility test, and reticulocyte results were all negative, while a peripheral blood smear revealed rouleaux formation, indicating endothelial injury. Autoimmune disease was tentatively diagnosed by antinuclear antibody (ANA) titer positivity (1:1,280), a high erythrocyte sedimentation rate (ESR; 92 mm/h), and low C3 and C4 levels (C3, 45.6 mg/dL; C4, 2.68 mg/dL) as well as negative anti-mitochondrial and smooth muscle Ab test results.

**Figure 1:**
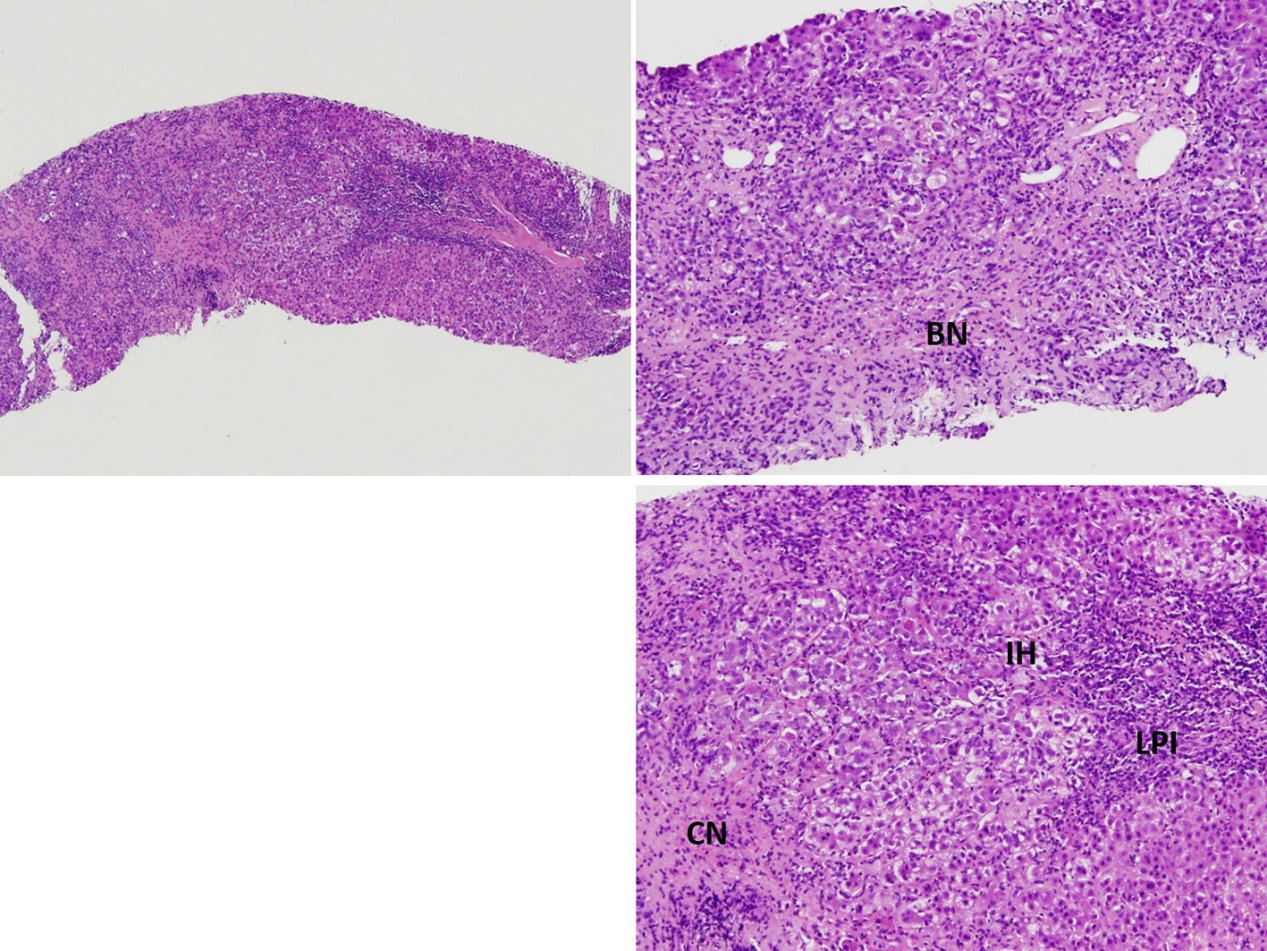
Pathological findings of the liver biopsy showed marked lymphoplasmocytic infiltration (LPI), bridging necrosis (BN) and confluent necrosis (CN), and prominent interface activity (IH), findings compatible with autoimmune hepatitis.

A liver biopsy was performed to screen for AIH. However, the patient developed a fever of 39.2°C after the liver biopsy for which antibiotic therapy of cefazolin was administered. A generalized maculopapular skin rash developed with itchiness at the upper extremities after the administration of acetaminophen. An allergy to acetaminophen was suspected, and the rash subsided spontaneously after its discontinuation. However, a bilateral reddish-purplish facial rash with a butterfly pattern developed 1 day later. This facial malar rash persisted during the hospitalization period. Positive anti-ds DNA Ab (242 WHO unit/mL) and IgG 4080 (mg/dL) were reported, while anti-phospholipid Ab and anti-cardiolipin Ab test results were negative. The pathological report of the liver biopsy showed features of lobular hepatitis with marked lymphoplasmacytic infiltration, bridging and confluent necrosis, and prominent interface activity, which were compatible with AIH (Figure 1). Albumin was infused for 3 days to correct the hypoalbuminemia (1.92 g/dL). Antibiotic therapy with ceftazidime was administered in response to the positive blood culture of *Acinetobacter* and the fever episodes gradually subsided. As the patient met the type I AIH and SLE Systemic Lupus Collaborating Clinics (SLICC) diagnostic criteria (1), intravenous methylprednisolone 40 mg Q12H was administered and then tapered according to the liver function test results and bilirubin level, followed by oral prednisolone 50 mg/day. Follow-up lab data after a 5-day-course of methylprednisolone showed improved liver enzymes (AST, 160 U/L; ALT, 226 U/L); serum IgG (2,990 mg/dL); total bilirubin, 6.3 mg/dL (direct bilirubin, 3.96 mg/dL); and anti-dsDNA Ab level (201.3 WHO unit/mL). She was discharged with satisfactory clinical remission.

The steroid-sparing agent, hydroxychloroquine 200 mg QD was administered to treat SLE since the first pediatric rheumatic outpatient clinic follow-up. Over 2 months, moon face developed and her body weight increased from 55 to 66 kg, and another steroid-sparing agent azathioprine 50 mg/day was administered after 6 weeks of steroid treatment in parallel with the decreasing ESR and remission of the facial malar rash. The prednisolone was gradually decreased to a maintenance dose of 5 mg/day in 3 months. An 80% reduction in transaminase levels was achieved by 4 weeks of treatment, while the liver function test results were completely normalized in 2 months. The patient's condition remained stable during the last 3 years of outpatient clinic follow-up without relapse. No renal or central nervous system involvement of the SLE was noticed. However, after a 3-year course of treatment, remission of the AIH was not achieved (ANA titer, 1:80). The liver function test results remained in the normal range (AST, 14 U/L; ALT, 7 U/L). Other rheumatic laboratory workups showed a normal complete blood cell count, elevated ESR (40 mm/h), anti-ds DNA Ab (<40.5 WHO unit/mL), normal complement levels (C3 120.0 and C4 15.5 mg/dL). Abdominal sonography showed no evidence of liver fibrosis or cirrhosis. Her maintenance medication included prednisolone 5 mg QOD, azathioprine 50 mg QOD, and hydroxychloroquine 200 mg QOD.

## Discussion

Once a patient meets the newest SLICC criteria (1) for SLE and International Autoimmune Hepatitis Group scoring for AIH, AIH–SLE overlap syndrome should be considered. According to the American Association for the Study of Liver Diseases practice guidelines, a revised original scoring system of the International Autoimmune Hepatitis Group for the diagnosis of AIH was established (5, 7). In our case, AIH was diagnosed based on the aggregate scores including female sex (+2), ALP:AST (or ALT) ratio <1.5 (136:931 or 136:507) (+2), elevated serum globulin or IgG (> 2.0 ×) (+3), ANA, SMA (anti-SM Ab) or liver kidney microsome type 1 > 1:80 (+3), negative hepatitis viral markers (+3), negative drug history (+1), average alcohol intake <25 g/day (+2), and histological features of interface hepatitis (+3). The pre-treatment aggregate score was 19 total points, which indicated a definite diagnosis, and 21 total points for the post-treatment aggregate score (+2) because of complete treatment response). According to serology, AIH is further subdivided into 2 types: type 1 positive for anti-nuclear antibody (ANA) and/or antismooth muscle antibody (SMA), while AIH-2 is positive for antiliver kidney microsomal antibody type 1 (anti-LKM1) and/or anti-liver cytosol type 1 (anti-LC1) (8, 9).

Classification as having SLE by the SLICC criteria (1) requires that a patient satisfy at least 4 of 17 criteria, including at least 1 of the 11 clinical criteria and one of the six immunologic criteria, or biopsy-proven lupus nephritis in the presence of antinuclear antibodies (ANA) or anti-double-stranded DNA (dsDNA) antibodies. Our patient met 4 criteria of the SLICC criteria for SLE proposed in 2012 by the Systemic Lupus Collaborating Clinics (1), including acute cutaneous lupus erythematosus, positive ANA titer, positive anti-dsDNA Ab on 2 occasions, and low complement level (C3, C4, or CH50). Therefore, the diagnosis of AIH–SLE overlap syndrome was established.

The therapeutic management of SLE is highly individualized and is based on clinical condition, including predominant disease manifestations, disease activity and severity, organ involvement, and previous treatment response. Among patients with SLE with any degree and type of disease activity, administration of hydroxychloroquine or chloroquine was suggested, unless these agents are contraindicated (10, 11). The benefits of hydroxychloroquine or chloroquine in SLE include relief of musculoskeletal manifestations, constitutional symptoms, and mucocutaneous manifestations (10). Also, hydroxychloroquine is steroid-sparing agent. Additional therapy, such as non-steroidal anti-inflammatory drugs, or glucocorticoids, is based upon the severity of disease and the combination of manifestations.

Overlap syndrome was usually used in the context of overlap of autoimmune hepatitis with primary sclerosing cholangitis (PSC) or primary biliary cholangitis (PBC), also called autoimmune sclerosing cholangitis (ASC). Although the case number is few, overlapping case of SLE and AIH has been occasionally diagnosed and reported (12, 13). Pediatric sclerosing cholangitis also has autoimmune features similar to AIH type 1, and the differential diagnosis depends on cholangiography (8). The co-occurrence of AIH and SLE is rare, especially in the pediatric population (3, 14). The reveal prevalence of overlapping SLE and AIH still remained unknown. Balbi et al. (15) reported in multicenter cohort study that AIH in pediatric SLE patients confirmed by biopsy was observed in 7/847 (0.8%) and all were diagnosed during adolescence. The majority occurred before or at SLE diagnosis [5/7 (71%)]. Irving et al. (16) reported incidence of AIH is 11.6% (42.1% of the patients with GIS involvement), and the incidence of AIH was significantly higher in children than adults (9.8 vs. 1.3%; *p* < 0.001). A case series and review of the literature by Beisel et al summarized previous cases of overlap syndrome (17). All of the pediatric cases reported to date including ours are summarized in Table 1.

**Table 1:** Summary of pediatric case reports in literature.

**References**	**Age**	**Sex**	**Clinical presentation**	**Treatment**	**Time of follow-up**	**Outcomes**
Mackay et al. (18)	16	F	Failure to thrive, jaundice, non-erosive arthritis, oral aphthous lesions	Cortisone	Not reported	Progression
Usta et al. (3)	12	F	Jaundice, hepatosplenomegaly, polyarthralgia, malaise, arthritis, butterfly-type facial erythema	Prednisolone Hydroxychloroquine Chloroquine	3 years	Stationary
Deen et al. (19)	13	M	Articular involvement, cardiopulmonary involvement	Prednisolone Azathioprine Chloroquine	Not reported	Remission
	13	F	Splenomegaly, articular involvement	Prednisolone Azathioprine Chloroquine	Not reported	Remission
	17	F	Jaundice, ascites, cutaneous involvement, proteinuria > 0.5 g/day, cardiopulmonary involvement	Prednisolone Azathioprine Chloroquine	Not reported	Remission
	11	F	Jaundice, cutaneous involvement, articular involvement, proteinuria > 0.5 g/day, cardiopulmonary involvement	Prednisolone Azathioprine Chloroquine	Not reported	Remission
Lai et al. (2015)	16	F	Jaundice, cholestatic hepatitis, malaise, butterfly-type facial erythema	Prednisolone Azathioprine Hydroxychloroquine	3 years	Remission
Battagliotti et al. (13)	16	F	Jaundice, renal and articular involvement, butterfly-type facial erythema	Prednisolone Mycophenolate mofetil	2 years	Remission

*Revised from the Table 1 of Beisel et al. (17)*.

SLE patients have a 25–50% chance of developing abnormal liver test function in their lifetime, and the most common causes include hepatotoxic drug use, coincident viral hepatitis, nonalcoholic fatty liver disease, hepatic arteritis, and nodular regenerative hyperplasia (20, 21). However, AIH is an autoimmune disease with liver inflammation whose exact cause remains unclear.

Lupus hepatitis and AIH–SLE overlap syndrome have not been clearly differentiated due to similarities in their clinical and biochemical features; however, this is important since their complications and therapies differ. Compared to hepatitis in the setting of SLE, AIH–SLE overlap syndrome has a more aggressive histological pattern and untreated AIH has a poor prognosis (5-year survival rate, 50%; 10-year survival rate, 10%). High-dose prednisone (1–2 mg/kg daily) is administered for up to 2 weeks to treat AIH-SLE overlap syndrome, while lupus hepatitis is treated with non-steroidal anti-inflammatory drugs, corticosteroids, and immunomodulators (3, 22).

Due to its numerous similarities with lupus hepatitis, AIH was previously called lupoid hepatitis, while histological examination of the liver shows specific changes in AIH. In AIH, the typical histological feature of AIH is interface hepatitis, characterized by a dense inflammatory infiltrate composed of lymphocytes and plasma cells, which crosses the limiting plate and invades the surrounding parenchyma (9). Other common histological finding include panlobular hepatitis with bridging necrosis (9). Some features may also suggest the diagnosis of AIH is emperipolesis and liver cell rosette formation (9, 18). These features appear in AIH but not lupus hepatitis. In contrast to AIH, lupus hepatitis presents as lobular hepatitis, atrophy and necrosis of the central hepatic cells, fatty infiltration, and an inflammatory infiltrate consisting mainly of lymphocytes. Lupus hepatitis patients frequently have a positive test for ribosomal P antibody (21, 23), and there appears to be an association of antiribosomal P antibody and lupus hepatitis (24). In short, AIH has a more aggressive histological pattern than the majority of cases of hepatitis in SLE (25). The liver biopsy in our case revealed prominent interface activity, acute lobular hepatitis with bridging and confluent necrosis, and an inflammatory infiltrate consisting of lymphocytes and few plasma cells, which support the diagnosis of AIH.

AIH–SLE overlap syndrome reportedly responds rapidly to steroid therapy and has a generally good prognosis (6). However, there have been no randomized controlled treatment trials in children with AIH, and very few reports have documented the efficacy of regimens similar to those used in adults (5). For children, prednisone is the preferred regimen and usually administered initially at a dose of 1–2 mg/kg daily (maximum 60 mg daily), but tapering schedules vary. Moreover, the early administration of azathioprine (1–2 mg/kg daily) or 6-mercaptopurine (1.5 mg/kg daily) for all children without contraindications is also recommended because of the significant deleterious side effects of long-term intermediate- or high-dose corticosteroid therapy on linear growth, bone development, and physical appearance (5). In 2016, Czaja presented new treatment guidelines for AIH in which prednisone or a combination of prednisolone and azathioprine is the mainstay of therapy (4). Mieli-Vergani (8) reported in 2018 that the conventional treatment of AIH consists of prednisolone, and the timing for the addition of azathioprine as a steroid-sparing agent varies according to the protocol used in the different centers. Approximately, 85% of the patients eventually require the addition of azathioprine despite using which kind of protocol. Budesonide, mycophenolate mofetil, and calcineurin inhibitors can also be considered in selected patients as frontline or salvage therapies. Our patient responded very well to corticosteroid therapy (initial dose, 1 mg/kg/day), and the initial intravenous methylprednisolone 40 mg Q12H for 4 days was soon tapered to oral prednisone 1 mg/kg/day.

Martínez Casas et al. (26) reported that adult patients with AIH-PBC had greater progression to cirrhosis (22.2 vs. 13.1%, *p* = 0.038), even in those who achieved partial or complete biochemical remission without relapse. Also these adult patients had greater indication of orthotopic liver transplantation (*p* = 0.009), but no differences in mortality. However, Rodrigues et al. (27) reported statistically significant difference of prognosis and response to treatment was observed between the pediatric AIH and ASC groups.

According to previous studies of overlap syndrome, disease relapse is common, and long-term low-dose prednisone or azathioprine therapy remains the treatment of choice after multiple relapses (3). After previous relapse and retreatment, 28% of patients can achieve a treatment-free state (28). Only a small minority of patients will progress to cirrhosis and require liver transplantation. Despite the disease severity at presentation, the response to corticosteroids with or without azathioprine is generally excellent in children (3, 5). However, there seems to be a non–relapsing association in the case reports of AIH-SLE overlapping in the recent years. Sönmez et al. (12) reported 8 pediatric SLE-AIH overlapping patients with good response to immunosuppressive treatment and good prognosis of AIH. Since the case number is still few, further discussion should be done if more cases reported in the future.

## Conclusion

For children with SLE and impaired liver function, screening for AIH should be considered because these two can occur together as overlap syndrome. Vice versa, Overlapping of SLE and AIH should be suspected when AIH patients present with a malar or other skin rash. Liver biopsy plays an important role in establishing the differential diagnosis of SLE with liver impairment or overlap with AIH. The prompt diagnosis and adjustment of further treatment plans can improve disease outcomes and prevent liver disease progression.

## Ethics Statement

This patient and her parents provided all of the clinical and laboratory information and samples and agreed to the case publication.

## Consent to Publish

Written informed consent was obtained from the patient for publication of this case report and any accompanying images.

## Author Contributions

W-TL and W-HC drafted the initial manuscript, designed the study, interpreted the data, screened the literature, and approved the final manuscript for submission. W-TL and W-HC are equal contributors in this study. H-LE interpreted the specimen and provided recommendations for this manuscript. M-HK provided recommendations for this manuscript. F-CH conceptualized the study, reviewed and revised the manuscript, and approved the final manuscript for submission. All authors approved the final manuscript as submitted and agree to be accountable for all aspects of the work.

### Conflict of Interest Statement

The authors declare that the research was conducted in the absence of any commercial or financial relationships that could be construed as a potential conflict of interest.

## Abbreviations

SLE: systemic lupus erythematosus
AIH: autoimmune hepatitis
ALP: alkaline phosphatase
AST: aspartate aminotransferase
ALT: alanine aminotransferase
ANA: antinuclear antibody
anti-dsDNA: anti-double-stranded DNA
GGT: gamma-glutamyl transpeptidase
PT: prothrombin time
aPTT: activate partial thromboplastin time
IgM: immunoglobulin M
Ab: antibody
IgG: immunoglobulin G
TIBC: total iron binding capacity
PB: peripheral blood
RBC: red blood count
ESR: erythrocyte sedimentation rate
C3: Complement component 3
C4: Complement component 4
SMA: anti-smooth muscle antibody
anti-LKM1: antiliver kidney microsomal antibody type 1
anti-LC1: anti-liver cytosol type 1
PSC: primary sclerosing cholangitis
PBC: primary biliary cholangitis
ASC: autoimmune sclerosing cholangitis.
