# Corrigendum: Overlap Syndrome Involving Systemic Lupus Erythematosus and Autoimmune Hepatitis in Children: A Case Report and Literature Review

**DOI:** 10.3389/fped.2019.00541

**Published:** 2020-01-24

**Authors:** Wan-Tz Lai, Wan-Hua Cho, Hock-Liew Eng, Ming-Hui Kuo, Fu-Chen Huang

**Affiliations:** ^1^Department of Pediatrics, Kaohsiung Chang Gung Memorial Hospital, Chang Gung University College of Medicine, Kaohsiung, Taiwan; ^2^Department of Ophthalmology, Kaohsiung Chang Gung Memorial Hospital, Chang Gung University College of Medicine, Kaohsiung, Taiwan; ^3^Department of Anatomic Pathology, Kaohsiung Chang Gung Memorial Hospital, Chang Gung University College of Medicine, Kaohsiung, Taiwan

**Keywords:** overlap syndrome, childhood, systemic lupus erythematosus, autoimmune hepatitis, jaundice

In the original article, there was an error. We neglected to include a statement and indication that the first two authors contributed equally to the work.

A correction has therefore been made to the **Author Contributions** statement and a symbol has been added in the author list.

“W-TL and W-HC drafted the initial manuscript, designed the study, interpreted the data, screened the literature, and approved the final manuscript for submission. W-TL and W-HC are equal contributors in this study. H-LE interpreted the specimen and provided recommendations for this manuscript. M-HK provided recommendations for this manuscript. F-CH conceptualized the study, reviewed and revised the manuscript, and approved the final manuscript for submission. All authors approved the final manuscript as submitted and agree to be accountable for all aspects of the work.”

The authors apologize for this error and state that this does not change the scientific conclusions of the article in any way. The original article has been updated.

